# Planteose as a storage carbohydrate required for early stage of germination of *Orobanche minor* and its metabolism as a possible target for selective control

**DOI:** 10.1093/jxb/erv116

**Published:** 2015-03-28

**Authors:** Takatoshi Wakabayashi, Benesh Joseph, Shuhei Yasumoto, Tomoyoshi Akashi, Toshio Aoki, Kazuo Harada, Satoru Muranaka, Takeshi Bamba, Eiichiro Fukusaki, Yasutomo Takeuchi, Koichi Yoneyama, Toshiya Muranaka, Yukihiro Sugimoto, Atsushi Okazawa

**Affiliations:** ^1^Department of Biotechnology, Graduate School of Engineering, Osaka University, 2-1 Yamadaoka, Suita, Osaka 565-0871, Japan; ^2^Department of Applied Biological Science, Nihon University, 1866 Kameino, Fujisawa, Kanagawa 252-8510, Japan; ^3^Applied Environmental Biology, Graduate School of Pharmaceutical Science, Osaka University, 1-6 Yamadaoka, Suita, Osaka 565-0871, Japan; ^4^International Institute of Tropical Agriculture Kano, PMB3112, Sabo Bakin Zuwo Road, Kano, Nigeria; ^5^Weed Science Center, Utsunomiya University, 350 Mine-machi, Utsunomiya 321-8505, Japan; ^6^Department of Biofunctional Chemistry, Graduate School of Agricultural Science, Kobe University, 1-1 Rokkodai, Nada-ku, Kobe 657-8501, Japan; ^7^Department of Applied Life Sciences, Graduate School of Life and Environmental Sciences, Osaka Prefecture University, 1-1 Gakuen-cho, Naka-ku, Sakai, Osaka 599-8531, Japan

**Keywords:** Broomrapes, metabolomics, nojirimycin, planteose, root parasitic weeds, seed germination, selective control, sugar metabolism.

## Abstract

Root parasitic weeds in Orobanchaceae cause serious damage to worldwide agriculture. Germination of the parasites requires host-derived germination stimulants, such as strigolactones, as indicators of host roots within reach of the parasite’s radicles. This unique germination process was focused on to identify metabolic pathways required for germination, and to design a selective control strategy. A metabolomic analysis of germinating seeds of clover broomrape, *Orobanche minor*, was conducted to identify its distinctive metabolites. Consequently, a galactosyl-sucrose trisaccharide, planteose (α-d-galactopyranosyl-(1→6)-β-d-fructofuranosyl-(2→1)-α-d-glucopyranoside), was identified as a metabolite that decreased promptly after reception of the germination stimulant. To investigate the importance of planteose metabolism, the effects of several glycosidase inhibitors were examined, and nojirimycin bisulfite (NJ) was found to alter the sugar metabolism and to selectively inhibit the germination of *O*. *minor*. Planteose consumption was similar in NJ-treated seeds and non-treated germinating seeds; however, NJ-treated seeds showed lower consumption of sucrose, a possible intermediate of planteose metabolism, resulting in significantly less glucose and fructose. This inhibitory effect was recovered by adding glucose. These results suggest that planteose is a storage carbohydrate required for early stage of germination of *O. minor*, and NJ inhibits germination by blocking the supply of essential glucose from planteose and sucrose. Additionally, NJ selectively inhibited radicle elongation of germinated seeds of Orobanchaceae plants (*Striga hermonthica* and *Phtheirospermum japonicum*). Thus, NJ will be a promising tool to develop specific herbicides to the parasites, especially broomrapes, and to improve our understanding of the molecular mechanisms of this unique germination.

## Highlight

Planteose metabolism was uncovered as a key metabolic pathway for germination of *Orobanche minor*. Inhibition of planteose metabolism by nojirimycin resulted in selective inhibition of *O. minor* germination.

## Introduction

Root parasitic weeds in Orobanchaceae are among the most destructive agricultural weeds. Within this family, *Orobanche* spp., *Phelipanche* spp., and *Striga* spp. cause particularly devastating damage to agricultural crops worldwide. The holoparasitic broomrapes, *Orobanche* spp. and *Phelipanche* spp., are mainly distributed in the Mediterranean region, Southern and Eastern Europe, and West Asia, and cause damage to a wide range of vegetables, beans, and other agricultural crops. The hemiparasitic witchweeds, *Striga* spp., which are mainly distributed in Africa, are thought to be the largest biological cause of serious crop losses on the continent. *Striga* is estimated to cause losses of at least US$7 billion per year (Elzein and Kroschel, 2003; Aly, 2007; Heide-Jørgense, 2008; Parker, 2009). These root parasitic weeds have evolved many parasitic adaptations; therefore, they have unique life cycles that are tightly coupled with the ecological behaviours of the host plants. For example, the seeds of root parasitic weeds in Orobanchaceae require host-derived germination stimulants, such as strigolactones, to germinate (López-Ráez *et al.*, 2009; Yoneyama *et al.*, 2009). Recently, strigolactones have been shown to induce hyphal branching in arbuscular mycorrhizal fungi (Akiyama *et al.*, 2005), and to function as a hormone to inhibit the branching of plant shoots (Gomez-Roldan *et al.*, 2008; Umehara *et al.*, 2008). Further studies have revealed that strigolactones might play a role in optimizing plant growth and development to cope with limiting resources (Brewer *et al.*, 2013). Seeds of root parasitic weeds usually require a period of imbibition for several days at suitable temperatures (a preparatory step known as ‘conditioning’) before they can respond to germination stimulants (Logan and Stewart, 1992). After germination, the radicles of root parasitic weeds attach to the host roots via specialized parasitic organs known as haustoria, and draw away water and nutrients from the hosts, causing serious reductions in crop growth and yield. The mature flowers of root parasitic weeds produce numerous, tiny, long-lived seeds. The complex life cycle of root parasitic weeds and their close association with the host plants make conventional weed control strategies virtually ineffective (Joel *et al.*, 1995, 2007). In addition, root parasitic weeds have often caused irreversible damage by the time the infestation is detected from the above ground emergence of their shoots.

Several methods for controlling root parasitic weeds have been proposed, and a chemical control method is one of the practical strategies (Hearne, 2009). Herbicides can control root parasitic weeds to some extent (Aly, 2007; Rubiales and Fernández-Aparicio, 2012). For example, glyphosate, an inhibitor of 5-enolpyruvylshikimate-3-phosphate synthase, and imidazolinones and sulfonylureas, inhibitors of acetolactate synthase, are used to control root parasitic weeds. However, these herbicides are not fully selective for root parasitic weeds and may damage host plants. To avoid damaging the host, the most effective strategy is to reduce the soil seed bank and/or inhibit the parasite at an early growth stage (e.g. germination and radicle elongation). In that sense, ‘suicidal germination’ is one of the most attractive strategies to reduce the soil seed bank (Zwanenburg *et al.*, 2009). In this approach, the seeds of the parasite are forced to germinate by applying a natural or synthesized germination stimulant, generally a strigolactone, to fields without a host crop, resulting in the death of the root parasitic weed. While this is a promising approach, it is expensive because it requires large-scale synthesis of structurally complex germination stimulants. Also, application of strigolactones could have adverse environmental effects, because they function as hormones and/or signalling molecules in plants and soil fungi. As an alternative, inhibitors targeting the early growth stages of root parasitic weeds have been screened, and many such natural compounds from fungi have been found (Vurro *et al.*, 2009). Some compounds have been shown to strongly inhibit seed germination and radicle elongation; for example, some macrocyclic trichothecenes inhibited seed germination of *Orobanche* (*Phelipanche*) *ramosa* at 0.1 μM. However, their mode(s) of action (MOA) is unknown, and their negative effects on the growth of other organisms (e.g. host plants or environmental microorganisms) have not been fully evaluated. In this context, the research presented here focuses on the unique germination process of root parasitic weeds to identify novel metabolic targets, which could be used to develop a selective control method. If these seeds have a specific metabolic process that is essential for germination, then inhibitors of that process could specifically inhibit germination without affecting the hosts or other organisms.

Metabolomics has proved to be a powerful technology in identifying the MOA of bioactive compounds (Aliferis and Chrysayi-Tokousbalides, 2010; Aliferis and Jabaji, 2011), in identifying novel metabolic pathways, and in evaluating in detail the cellular responses of plants (Weckwerth and Fiehn, 2002). Here, a metabolomics approach was used to identify potential targets for the selective control of root parasitic weeds. Gas chromatography combined with time-of-flight mass spectrometry (GC-TOF-MS) was used to evaluate the metabolomic profiles of germinating seeds of clover broomrape (*Orobanche minor*). This provided data on the primary metabolism of these seeds, and allowed identification of the distinctive aspects of sugar metabolism during germination. The effects of several glycosidase inhibitors on sugar metabolism during *O*. *minor* seed germination were analysed. Nojirimycin bisulfite (NJ) suppressed sugar metabolism, resulting in the selective inhibition of seed germination of *O*. *minor*.

## Materials and methods

### Germination assay

The seed germination assay for parasitic weeds was conducted as described previously (Chae *et al.*, 2004), with some modifications. Seeds of root parasitic weeds were surface-sterilized with a solution containing 1% (v/v) sodium hypochlorite and 0.1% (v/v) Tween 20 for 2min, rinsed with distilled water, and dried under vacuum. Approximately 50 surface-sterilized seeds were conditioned on a filter disk (10mm, Whatman GF/D; GE Healthcare Bio-Sciences AB, Uppsala, Sweden) placed on another filter paper (47mm, Whatman GF/D) in a Petri dish (50mm) with 1.5mL distilled water, and kept in the dark at 23°C for 1 week (*O*. *minor*, *Orobanche crenata*, and *Phelipanche aegyptiaca*) or 2 weeks (*Striga hermonthica*). Four filter disks with seeds were placed in each Petri dish. After conditioning, the GF/D disks with the seeds were transferred to a new Petri dish containing fresh filter paper (47mm, Whatman GF/D) to remove surplus water. Germination was induced by adding 1.5mL GR24 solution [0.1–1.0 mg·L^-1^ (w/v)]. After the germination stimulation, seeds were observed under a microscope to count germinated seeds. The germinating seeds were stained with crystal violet to facilitate detection of radicles if necessary.

*Phtheirospermum japonicum* is a facultative hemiparasite closely related to *Orobanche* and *Striga* (Bennett and Mathews, 2006). The seeds were surface-sterilized, vernalized at 4°C for 2 days, and then incubated at 25°C in the dark. The root length was measured 5 days after imbibition.

Seeds of planteose-containing plants, tomato (*Solanum lycopersicum*) (Amuti and Pollard, 1977; Gurusinghe and Bradford, 2001), sesame (*Sesamum indicum*) (Hatanaka, 1959), and spearmint (*Mentha spicata*) (French *et al.*, 1959) were surface-sterilized, vernalized at 4°C for 2 days, and then incubated at 23°C under a 16h light/8h dark photoperiod (sesame and spearmint), or at 25°C in the dark (tomato). Root lengths of tomato and sesame seeds were measured 4 days after imbibition, and those of spearmint 10 days after imbibition.

### Glycosidase inhibitor assay

The effects of glycosidase inhibitors NJ (Niwa *et al.*, 1970; Reese *et al.*, 1971; Dale *et al.*, 1985; Kodama *et al.*, 1985), castanospermine (CS) (Saul *et al.*, 1983), 1-deoxynojirimycin (DNJ) (Saunier *et al.*, 1982) and 1-deoxygalactonojirimycin (DGJ) (Legler and Pohl, 1986) were tested by mixing them with GR24 solution. For the germination recovery test, sugar or uridine diphosphate (UDP)-glucose (final concentration 10mM) was mixed with GR24 (final concentration 1.0 mg·L^-1^) and NJ (final concentration 10 µM). Radicle and root lengths were measured using ImageJ 1.47v software (http://rsbweb.nih.gov/ij/).

### Sample preparation for metabolomics, sugar analysis, and protein extraction

For metabolomic analysis, sugar analysis, and protein extraction, 50–100mg of seeds of *O. minor* were conditioned on two layers of filter paper (47mm, Whatman GF/D) in a Petri dish (50mm) with 1.5mL distilled water at 23°C in the dark for 1 week. Germination and NJ treatments were conducted as described above. Samples were collected at various times during conditioning and after the GR24 treatment, and were stored at −80°C until use.

Seeds of *O*. *crenata*, *P*. *aegyptiaca*, and *S*. *hermonthica* were collected at different days because their germination rates were different. Seeds of *Striga* can germinate faster than those of *Orobanche* and *Phelipanche* (Wigchert *et al.*, 1999; Matusova *et al.*, 2005).

### GC-TOF-MS analysis

Metabolite extraction, derivatization, and GC-TOF-MS analysis were conducted as described previously (Jumtee *et al.*, 2009). Peak deconvolution, identification, and quantification were performed using the Pegasus software package ChromaTOF ver. 2.32 (LECO Corp., St. Joseph, MI, USA). The principal component analysis (PCA) was carried out with the data set obtained from metabolic profiling using Pirouette software (Informetrix, Inc., Bothell, WA, USA) as described previously (Jumtee *et al.*, 2008, 2009). The amount of total fatty acids was calculated as the sum of the peak areas of different fatty acids detected by GC-TOF-MS.

### Sugar analysis with GC-MS

The frozen seeds were disrupted by ball milling (20 Hz, 2min) with an MM 301 mixer mill (Retsch GmbH, Haan, Germany) and then extracted in 300 μL distilled water at 95°C for 30min. The solution was centrifuged at 12,000 *g* for 10min, and the supernatant was collected in a new Eppendorf tube. Proteins were removed from the extract by ultrafiltration with an Amicon Ultra-0.5 10K centrifugal filter (Merck KGaA, Darmstadt, Germany). The solution was passed through a Chromatodisc filter (Type: 4A, pore-size: 0.2 μm; GL Sciences Inc., Tokyo, Japan) and then freeze-dried. The sample was dissolved in 100 μL pyridine, and an aliquot of the sample was derivatized with the same volume of *N*-trimethylsilylimidazole (Sigma-Aldrich, St. Louis, MO, USA) at room temperature.

GC-MS analysis was performed using a JMS-AMSUN200 quadrupole mass spectrometer (JEOL Ltd., Tokyo, Japan) coupled to a gas chromatograph (6890A; Agilent Technologies, Inc., Palo Alto, CA, USA) equipped with an HP-5MS capillary column (30 m × 0.25mm internal diameter, 0.25 μm film thickness, Agilent Technologies, Inc.). An analysis was carried out in the splitless mode with a 1-μL injection volume. The injector temperature was 250°C, and the helium gas flow rate through the column was 1 mL·min^-1^. The column temperature was held at 70°C for 2min, then raised by 10°C min^-1^ to 325°C and held at that temperature for 10min. The interface temperature and ion source temperature were set at 280°C and 250°C, respectively. ions were generated by a 70eV electron beam, and two scans per second were recorded in the mass range of *m*/*z* 100–750. For each sample, chromatographic peaks were identified by comparing their retention time with those of authentic standards. Compounds were quantified from the peak areas using the external standard method.

### Purification of planteose

Sugars were extracted from dry seeds of *O. minor* as described above. The extract was concentrated by a centrifugal concentrator, and then the trisaccharide fraction was purified by isocratic high-performance liquid chromatography (HPLC) with a COSMOSIL Sugar-D column (20×250mm, 5 μm; Nacalai Tesque, Inc., Kyoto, Japan). Eluted compounds were detected with a Shimadzu RID-10A refractive index detector (Shimadzu Corp., Kyoto, Japan). The mobile phase was 65% acetonitrile. The column oven was set at 30°C, and the flow rate was 9.0 mL·min^-1^. HPLC was performed using an LC workstation (Shimadzu Corp.) with CLASS-VP ver. 6.1 software. The HPLC system consisted of a system controller (SCL-10Avp), a column oven (CTO-10A), an auto-sampler (SIL-10Axl), and a pump (LC-10AT).

### Nuclear magnetic resonance analysis

NMR spectra (^1^H, ^13^C, COSY, HMBC, HSQC, TOCSY, HSQC-TOCSY, and NOESY) of the purified trisaccharide were recorded on a JMN ECA-500 system (JEOL Ltd.) in D_2_O. The internal standard was 3-(trimethylsilyl)-propionic-2,2,3,3-*d*_4_ acid sodium salt.

### Sugar analysis by ultra-performance liquid chromatography and an evaporative light scattering detector

The dried sugar extracts were dissolved in 80% acetonitrile and analysed with the ACQUITY ultra-performance liquid chromatography (UPLC) system (Waters Corp., Milford, MA, USA) using a Waters ACQUITY UPLC BEH AMIDE column (2.1mm × 100mm, 1.7 μm). Eluted compounds were detected using a Waters evaporative light scattering detector (ELSD). The detector conditions were as follows: gain, 200; gas pressure, 50 psi (344.7 kPa); drift tube temperature, 55°C; nebulizer mode, cool. The mobile phase was acetonitrile with 0.2% triethylamine (solvent A) and distilled water with 0.2% triethylamine (solvent B). Separations were performed using a gradient programme with a mixture of solvents A and B, as follows: 20–30% B for 0–2.8min, 30–50% B for 2.8–4.5min, 50–80% B for 4.5–5.0min, 80–20% B for 5.0–6.5min, and 20% B for 6.5–7.0min (re-equilibration). The flow rate was set as follows: 0.25 mL·min^-1^ for 0–4.5min, 0.25–0.10 mL·min^-1^ for 4.5–5.0min, 0.10–0.25 mL·min^-1^ for 5.0–6.5min, and 0.25 mL·min^-1^ for 6.5–7.0min. The column oven was set at 35°C and the sample injection volume was 5 μL. For each sample, chromatographic peaks were identified by comparing the retention time with those of authentic standards. The chromatograms were analysed with the Waters Empower 2 data processing programme.

### Starch analysis

Starch was quantified with a starch HK assay kit (Sigma-Aldrich) according to manufacture’s instruction using ~50mg seeds.

### Invertase extraction and enzymatic assays

Protein extraction and the assays for activities of invertases (INVs) were conducted as described previously (Draie *et al.*, 2011), with some modifications. The frozen seeds were disrupted by ball milling (20 Hz, 2min) and extracted at 4°C in 1.5mL extraction buffer (pH 7.0) composed of 50mM HEPES, 1mM DTT, 1mM EDTA, 0.5 mg·mL^-1^ polyvinylpolypyrrolidone (PVPP), and 1% (v/v) protease inhibitor cocktail (Sigma-Aldrich). The homogenate was centrifuged at 12,000 *g* for 10min, and the supernatant was collected in a new Eppendorf tube. The pellet was re-extracted in extraction buffer without PVPP and the extract was collected in the same Eppendorf tube after centrifugation. The collected enzyme solution was used for the soluble acid invertase (SAI) and soluble neutral invertase (SNI) assays. The residue was rinsed with 1.0mL extraction buffer without PVPP and protease inhibitor cocktail, and the supernatant was discarded after centrifugation. To extract cell wall invertase (CWI), the pellet was extracted in extraction buffer containing 1.0M sodium chloride. After centrifugation, the supernatant was used for the CWI assay.

Before the assay, the enzyme extract was desalted on a PD-10 desalting column (GE Healthcare Bio-Sciences AB) previously equilibrated with 50mM HEPES buffer (pH 7.0), and then concentrated with an Amicon Ultra-15 10K centrifugal filter (Merck KGaA). The enzymatic reaction (200 μL reaction mixture) was initiated by mixing the extracted enzyme (20 μg) and sucrose (final concentration 100mM) in 50mM sodium phosphate buffer (pH 5.0 for SAI and CWI or PH 7.5 for SNI). When required, NJ was added to reaction mixture at a final concentration of 100 μM. The reaction mixture was incubated at 30°C for 2h, and the reaction was stopped by heating at 95°C for 3min. The amount of released glucose was determined using the Glucose (HK) Assay reagent (Sigma-Aldrich).

## Results

### Metabolic profiling of germinating *O. minor* seeds and identification of planteose

**Fig. 1.**
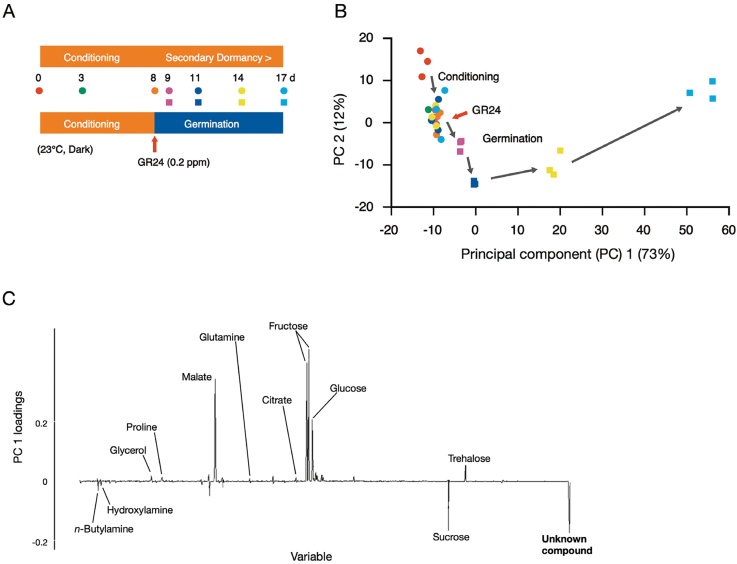
Metabolic profiling of germinating seeds of *O. minor*. **(A)** Squares (with GR24 treatment) or circles (without GR24 treatment) show time points of sample collection. GR24 was applied on day 8 (arrow). **(B)** PCA of metabolite profiles of seeds at different stages during conditioning and germination, shown as the combination of the first two PCs (representing 85% of metabolite variance). Each data point is an independent sample. **(C)** Loading plot showing weight for each data point of the total ion current chromatogram in calculating PC 1.

A metabolomic analysis of germinating seeds of *O*. *minor* was conducted to understand the metabolic changes during germination and to identify characteristic compound(s) required for germination. Germination was induced by the synthetic strigolactone, GR24, after 7 days of conditioning. The seeds were collected at various times during conditioning and germination (Fig. 1A), and derivatized hydrophilic metabolites and fatty acids were analysed by GC-TOF-MS. PCA of the GC-TOF-MS total ion current chromatograms provided a detailed view of the characteristic metabolic changes associated with the gradual transition of dormant seeds to germinating seeds via conditioning (Fig. 1B). In the PCA, principal component (PC) 1 accounted for 73% of the variation in metabolite content among samples during conditioning and germination. The loading of PC 1 indicated that the levels of amino acids, organic acids, and some sugars (glucose, fructose, and trehalose) increased during germination, whereas the levels of sucrose and an unknown compound decreased during germination (Fig. 1C). This unknown compound was predicted to be a trisaccharide, based on its retention time and mass spectrum. However, there was no corresponding spectrum in an in-house mass spectral library or in NIST MS library. The decreases in sucrose and the unknown trisaccharide implied that they play roles in the germination of *O. minor* seeds.

The unknown trisaccharide in the seeds was extracted from the dry seeds of *O. minor* and purified by HPLC (Supplementary Figure S1). GC-MS analysis of the acid hydrolysate of the purified trisaccharide, together with NMR analysis, identified the sample as planteose (α-d-galactopyranosyl-(1→6)-β-d-fructofuranosyl-(2→1)-α-d-glucopyranoside) (Supplementary Figs S2 and S3, and Supplementary Table S1).

### Changes in sugar contents during germination

**Fig. 2.**
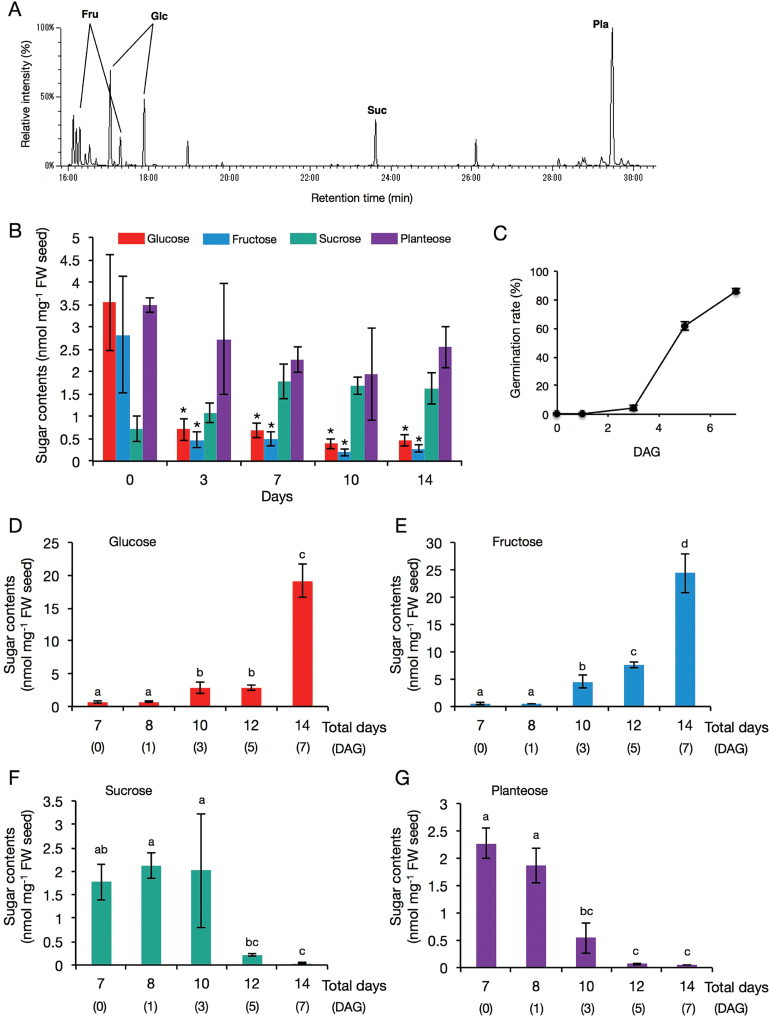
Sugar profiles in *O. minor* seeds during conditioning and germination. Galactose was not detected in any samples. **(A)** Total ion current chromatogram of sugars in dry seeds obtained by GC-MS analysis. Fru, fructose; Glc, glucose; Pla, planteose; Suc, sucrose. **(B)** Changes in the amounts of sugars in seeds without GR24 treatment (mean ± SD, *n* = 3). Conditioning period was 7 days and distilled water was applied on final day of conditioning, therefore germination was not induced. Asterisks indicate significant differences in each sugar contents between dry seeds and during conditioning seeds (*P* < 0.05, Tukey-Kramer). **(C)** Germination rate of *O. minor* seeds after GR24 treatment. Conditioning period was 7 days; 10 mg·L^-1^ (w/v) GR24 was applied on final day of conditioning. Seeds did not germinate without GR24 treatment. Seeds were observed and germinated seeds were counted under a microscope (mean ± SD, *n* = 3). **(D–G)** Changes in the amounts of sugars in seeds with GR24 treatment (mean ± SD, *n* = 3). Conditioning period was 7 days and 10 mg·L^-1^ (w/v) GR24 was applied on final day of conditioning. Different letters indicate significant differences in sugar contents during germination (*P* < 0.05, Tukey-Kramer).

Each sugar in *O. minor* seeds during germination was quantified by GC-MS analysis to investigate planteose metabolism (Fig. 2). In the dry seeds, the main sugars were planteose (3.48±0.17 nmol·mg^-1^ seeds), sucrose (0.73±0.29 nmol·mg^-1^ seeds), glucose (3.54±1.06 nmol·mg^-1^ seeds), and fructose (2.82±1.30 nmol·mg^-1^ seeds) (Fig. 2A). The amounts of glucose and fructose significantly reduced to about one-third shortly after imbibition, and the levels of these sugars—including sucrose and planteose—did not change for 2 weeks without GR24 treatment (Fig. 2B). After the GR24 treatment on the seventh day of imbibition, the germination rate of *O. minor* seeds gradually increased and reached a maximum rate 86.2% 7 days after GR24 treatment (DAG) (Fig. 2C). Planteose metabolism proceeded with a parallel increase in the amounts of glucose and fructose during germination (Fig. 2D–G). At 3 DAG, when the radicle had emerged, the planteose level began to decrease significantly, and the amounts of glucose and fructose gradually increased. The amount of sucrose significantly decreased from 5 DAG subsequent to the decrease of planteose (Fig. 2F and G). Planteose and sucrose were almost completely consumed by 5 DAG while germination rate increased drastically from 3 to 5 DAG (Fig. 2C, F and G). Galactose, a constituent of planteose, was not detected in any seed samples.

**Fig. 3.**
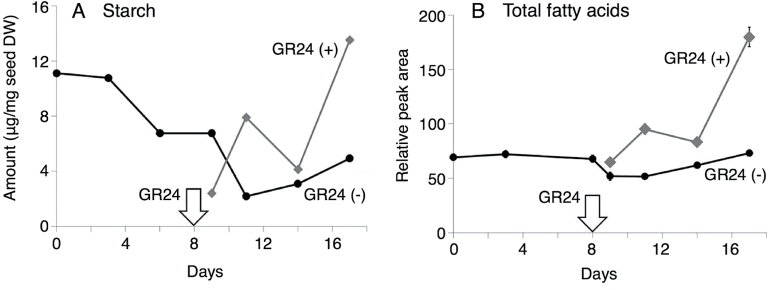
Profiles of starch and total fatty acids in germinating *O. minor* seeds. Amounts of **(A)** starch and **(B)** total fatty acids were measured in seeds with (+) or without (-) GR24 treatment (mean ± SD, *n* = 3). Arrows indicate the day of GR24 treatment.

The levels of starch and total fatty acids in *O. minor* seeds were also analysed to evaluate contributions of their metabolism to the germination. The amount of starch (10.4 µg·mg^-1^ dry weight) in the seeds of *O*. *minor* gradually decreased during conditioning, but increased again after GR24 treatment (Fig. 3A). The amount of total fatty acids in the dry seeds of *O. minor* increased after GR24 treatment and was greater than that in GR24 untreated seeds at any time points (Fig. 3B).

**Fig. 4.**
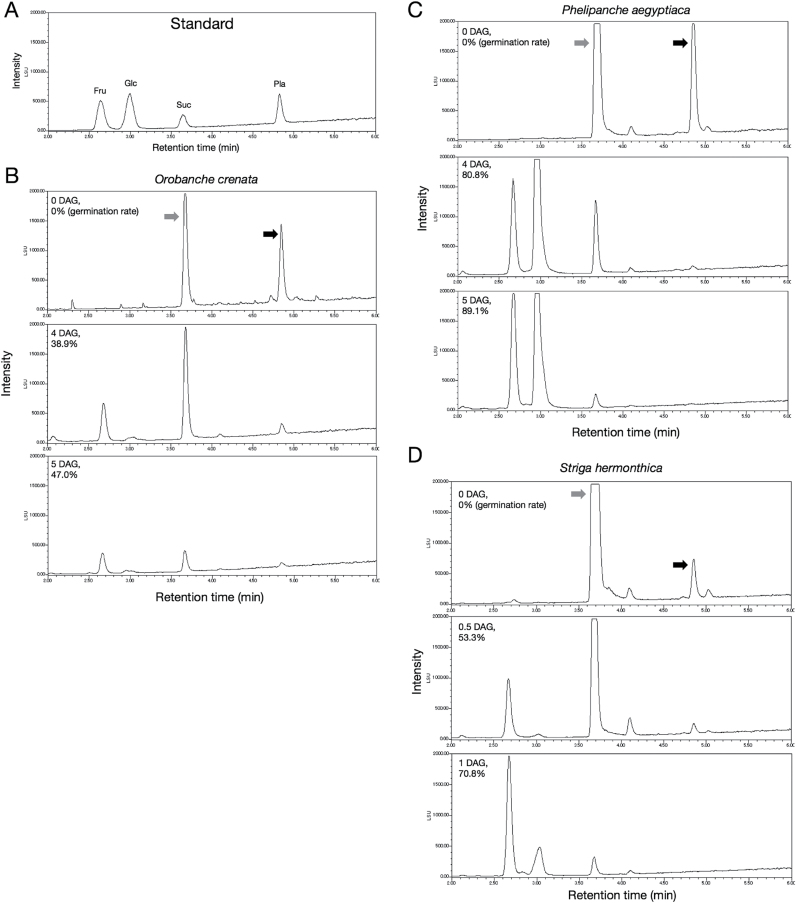
UPLC-ELSD analysis of sugars in seeds of various root parasitic weeds. **(A)** Authentic standard compounds. **(B)** *O*. *crenata*. **(C)** *P*. *aegyptiaca*. **(D)** *S*. *hermonthica*. Black and grey arrows indicate planteose and sucrose, respectively. DAG and germination rate at that time are shown in upper left of chromatograms. Seeds of each parasitic weed were collected immediately after conditioning and at two time points during germination before reaching maximum germination rate. The dates of collection of seeds were different among species because germination rate of each plant species is different.

Planteose was detected in the seeds of the other root parasitic weeds in Orobanchaceae, including *O*. *crenata*, *P*. *aegyptiaca*, and *S*. *hermonthica* (Fig. 4). The sugar compositions in seeds of other broomrapes (*O. crenata* and *P. aegyptiaca*) were similar to that in seeds of *O. minor*, and the amounts of planteose and sucrose (Fig. 4, black and grey arrows, respectively) decreased during germination. However, there was less planteose in *S. hermonthica* seeds compared with the seeds of the three broomrapes. The sucrose level in *S. hermonthica* seeds drastically decreased during germination, and there was a significant accumulation of monosaccharides at the later stage of germination, similar to those observed in the broomrapes.

### Effect of NJ, a glycosidase inhibitor, on germination and radicle elongation of root parasitic weeds

**Fig. 5.**
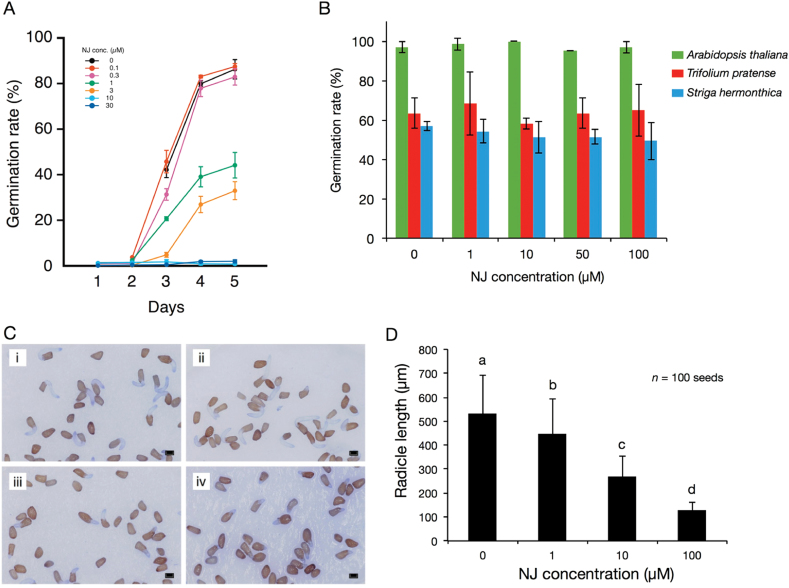
Effect of NJ on seed germination of parasitic and non-parasitic plants. **(A)** Effect of NJ at various concentrations on germination rates of *O. minor* seeds (mean ± SD, *n* = 3). Germination rate of *O. minor* seeds decreased in a dose-dependent manner with NJ. **(B)** Effect of NJ on seed germination rates in other plant species (mean ± SD, *n* = 3). **(C)** Morphological changes in radicle elongation of *S. hermonthica* in the presence of NJ. Staining by crystal violet facilitated visualization of the radicles. Treatments were as follows: (i) 10 mg·L^-1^ (w/v) GR24 only; (ii) GR24 + 1 μM NJ; (iii) GR24 + 10 μM NJ; and (iv) GR24 + 100 μM. Bar: 200 μm. **(D)** Radicle lengths of *S*. *hermonthica* treated with NJ at various concentrations. Radicle lengths of 100 germinating seeds were determined using ImageJ software. Radicle elongation was inhibited by NJ in a dose-dependent manner. Different letters indicate significant differences in radicle lengths (*P* < 0.05, Tukey-Kramer).

To determine the importance of planteose metabolism during germination, some glycosidase inhibitors were applied together with GR24. Among the tested inhibitors, NJ showed a strong and selective inhibitory effect on germination of *O. minor* seeds. NJ inhibited germination of *O. minor* seeds in a dose-dependent manner (Fig. 5A). NJ at 1 and 3 μM decreased the germination rate by 48.8% and 61.8%, respectively, and NJ at 10 μM or higher completely inhibited the seed germination (Fig. 5A). However, NJ had no effect, even at 100 μM, on seed germination of *Arabidopsis* and red clover (*Trifolium pratense*), a host of *O*. *minor* (Fig. 5B). In the case of *S. hermonthica*, NJ did not inhibit seed germination (Fig. 5B), but caused a dose-dependent reduction in radicle elongation (Fig. 5C and D). The radicle length of *S. hermonthica* with 10 µM and 100 μM NJ was nearly one-half and one-fifth of that of the control, respectively (Fig. 5D). To investigate whether NJ affects the growth of diverse plants in the Orobanchaceae, the effect of NJ on *P. japonicum* was analysed. Similar to the case in *S. hermonthica*, NJ did not affect the germination rate of *P. japonicum* seeds, but it inhibited root elongation at concentrations of 10 µM or higher (Supplementary Figure S4).

The glycosidase inhibitor CS inhibited *O*. *minor* seed germination at concentrations of 1 µM or higher; however, its inhibitory effect was weaker than that of NJ (reduction of 7.2% at 1 µM and 15.6% at 10 µM) (Supplementary Figure S5A). Germination was not affected by two other iminosugars, DNJ and DGJ, at concentrations up to 100 μM (Supplementary Figure S5B), but DNJ inhibited radicle elongation of *O. minor* (Supplementary Figure S6) in a similar way to NJ on *S*. *hermonthica*.

### Effect of NJ on planteose metabolism in *O. minor*

**Fig. 6.**
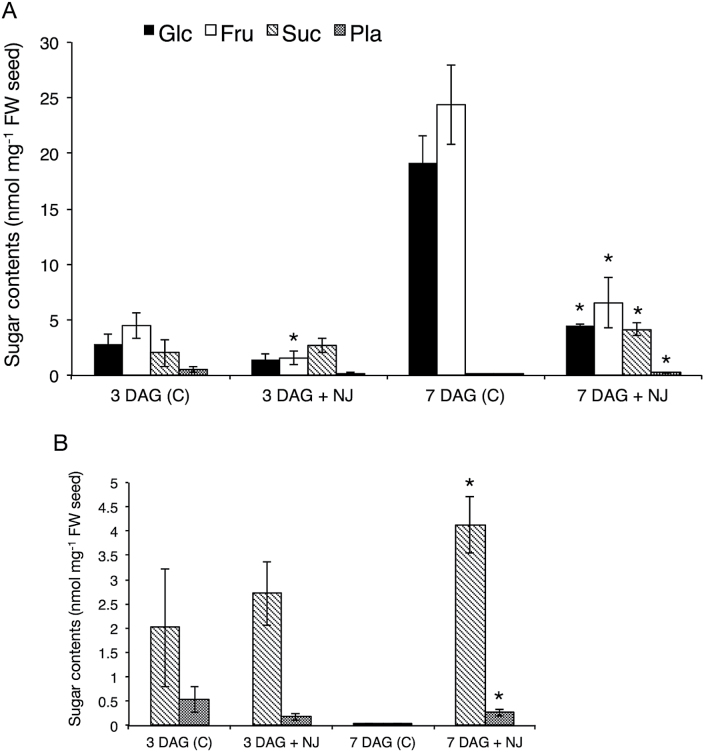
Changes in sugar contents in *O. minor* seeds in the presence of NJ. **(A)** Amounts of sugars in seeds at 3 DAG and at 7 DAG with (+NJ) or without (C, control) NJ treatment (mean ± SD, *n* = 3). NJ (10 μM) was added along with GR24 after the conditioning period. **(B)** Magnification of graph showing amounts of sucrose and planteose. Asterisks indicate significant differences in amounts of sugars between control and NJ-treated seeds on the same day (*P* < 0.05, Student’s *t* test). Fru, fructose; Glc, glucose; Pla, planteose; Suc, sucrose.

It was speculated that NJ causes changes in sugar metabolism in *O. minor* seeds, ultimately inhibiting their germination. To investigate the MOA of NJ, the sugars in germinating *O*. *minor* seeds in the presence of NJ were quantified. In seeds at 3 DAG, the sugar compositions were almost the same in non-treated and NJ-treated seeds. However, at 7 DAG, the NJ-treated seeds showed increased levels of sucrose and severely decreased levels of glucose and fructose compared with non-treated seeds (Fig. 6). The amounts of glucose and fructose in NJ-treated seeds were about one-quarter of those in non-treated seeds, and the amounts of sucrose and planteose in NJ-treated seeds were about 100 and five times higher than those in non-treated seeds, respectively. Hydrolysis of the glucose moiety of planteose can release planteobiose (α-d-galactopyranosyl-(1→6)-β-d-fructofuranose). Therefore, planteobiose is a possible intermediate in planteose metabolism, but this compound was not detected in any of the samples.

**Fig. 7.**
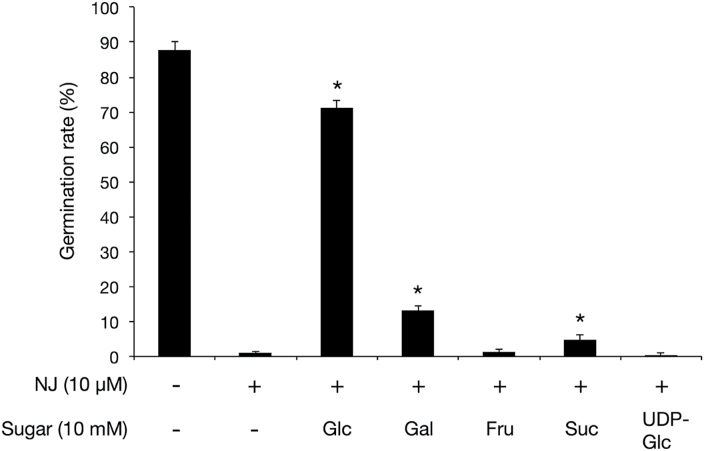
Recovery of seed germination rate by simultaneous addition of exogenous sugars and NJ. Seed germination rates were measured at 7 DAG (mean ± SD, *n* = 3). Asterisks indicate significant differences in germination rates between seeds treated with NJ and those treated with NJ + exogenous sugars (*P* < 0.05, Student’s *t* test). Fru, fructose; Gal, galactose; Glc, glucose; Pla, planteose; Suc, sucrose; UDP-Glc, UDP-glucose.

To investigate the relationship between the inhibitory effect of NJ on germination and sugar metabolism in *O. minor* seeds, exogenous sugars or a nucleotide sugar were applied together with GR24 and NJ. In plants, sucrose degradation is catalysed by INVs (EC 3.2.1.26, sucrose ↔ glucose + fructose) and sucrose synthases (SUSs) (EC 2.4.1.13, sucrose + UDP ↔ UDP-glucose + fructose). To determine whether the restricted supply of sucrose degradation products affects germination, UDP-glucose or the monosaccharides constituting planteose were added. The germination rate was significantly recovered to 71.2% by addition of glucose with NJ (Fig. 7). Exogenous galactose also recovered the germination rate of the NJ-treated seeds to 13.2%, but with a much lower efficiency than that of glucose. Fructose and UDP-glucose did not recover the germination rate of NJ-treated seeds. Sucrose recovered the germination rate to 4.8%; however, the degree of the recovery was significantly lower than that of glucose or galactose.

### Effect of NJ on activities of INV

**Fig. 8.**
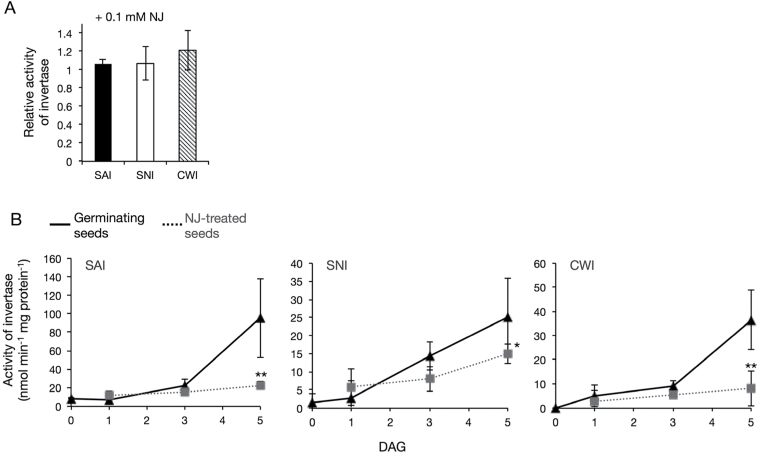
Effects of NJ on activities of INVs. **(A)** Effect of NJ on activities of INVs *in vitro*. Reaction mixture contained crude enzyme extract from germinating seeds at 5 DAG and NJ at a final concentration of 0.1mM. NJ effect was calculated as ratio of activity with NJ treatment to that without NJ (mean ± SD, *n* = 3). **(B)** Effect of NJ on activities of INVs *in vivo*. Crude enzyme extracts were prepared from germinating and 10 μM NJ-treated seeds of *O. minor*. Activities of SAI, SNI, and CWI were assayed. Solid and dashed lines show mean values of enzyme activity in non-treated and NJ-treated seeds, respectively (mean ± SD, *n* = 6 to 8). Enzyme activity is expressed as nmol glucose·min^-1^·mg protein^-1^. Asterisks indicate significant differences in enzyme activity between non-treated and NJ-treated seeds at same time point (* *P* < 0.05, ** *P* < 0.01, Student’s *t* test).

The results described above indicated that the inhibition of germination of *O. minor* by NJ is caused by the lack of an adequate supply of glucose. Therefore, the inhibition might be attributable to suppression of activities of INVs in the planteose metabolic pathway. In plants, INVs can be classified into three types according to their solubility, subcellular localization, pH optima, and isoelectric point. The SAIs localize in the vacuole, the SNIs localize in the cytosol, and CWIs are in the cell wall. These types of INVs were prepared as crude enzymes from germinating seeds of *O*. *minor* at 5 DAG. When NJ was added to the enzyme assays, it did not affect the activities of these INVs, even at 100 μM (Fig. 8A).

Next investigated were the changes in the activities of INVs over time during germination, and the effects of NJ on these activities. Crude enzyme extracts were prepared from germinating seeds and NJ-treated seeds. In germinating seeds, the activities of INVs increased during germination (Fig. 8B, solid line). The activities of INVs also gradually increased in NJ-treated seeds, but they were significantly lower in NJ-treated than in non-treated seeds at 5 DAG (Fig. 8B, dashed line). The activities of acid INVs (SAI and CWI) in NJ-treated seeds were about one-quarter those in germinating seeds. The activity of SNI was less affected by NJ treatment, and the activity in NJ-treated seeds was reduced by about 60% compared to that in non-treated germinating seeds. However, a comparable level of activity of α-galactosidase, which may catalyse the first step of planteose degradation, was observed in both non-treated and NJ-treated seeds (data not shown).

### Effect of NJ on germination of planteose-containing seeds of non-parasitic plants

These results indicated that planteose degradation might be involved in the early stage of germination, and that sucrose degradation and the supply of glucose after planteose degradation might be key steps in the seed germination of *O. minor*. NJ might inhibit seed germination in *O. minor* by interfering with activities of INVs, thereby inhibiting the degradation of sucrose in the planteose metabolic pathway. Therefore, NJ may also inhibit germination in seeds of other non-parasitic plants that contain the planteose metabolic pathway. To investigate the effects of NJ on planteose-containing seeds, we conducted germination assays and root length measurements using seeds of tomato, sesame, and spearmint. The germination rates of these seeds were not affected by NJ, even at 1mM (Supplementary Figure S7A). NJ inhibited root elongation of sesame and spearmint, but not tomato (Supplementary Figure S7B). However, the concentration of NJ required to inhibit root elongation was higher for sesame and spearmint than for *S. hermonthica* and *P. japonicum* (Fig. 5D, Supplementary Figure S4B). An analysis of NJ-treated and non-treated tomato seeds showed that they had the same sugar composition. In NJ-treated and non-treated tomato seeds, planteose was degraded, but the amount of sucrose remained constant throughout the germination process (Supplementary Figure S7C).

## Discussion

In the present study, the primary metabolism during *O. minor* seed germination has been examined using a metabolomics approach. The results show that planteose metabolism is involved in the early stage of germination. This is believed to be the first report of the presence of planteose in seeds of members of Orobanchaceae. Planteose was first identified in *Plantago* seeds and has since been found in the seeds of a number of other plants (Amuti and Pollard, 1977). It has been reported that planteose exists mainly in seeds, and that it functions as a storage carbohydrate because its amount increases during seed ripening and it is consumed during germination (Kandler and Hopf, 1982; Peterbauer and Richter, 2001; Downie *et al.*, 2003). However, it is still unknown whether planteose metabolism is essential for seed germination (Peterbauer and Richter, 2001). Planteose is an isomer of raffinose, a member of the raffinose family oligosaccharides (RFOs), which are composed of sucrose and chains of α-galactosyl residues attached to the glucose moiety of sucrose via an α-(1→6) galactosidic linkage. Planteose has an α-galactosidic linkage at the fructose moiety. In some plant species, RFOs might function as an easily available source of energy during the early stages of seed germination (Blöchl *et al.*, 2007). The most common RFO, raffinose, was not detected here in *O. minor* seeds. There was approximately five times more planteose than sucrose in the dry seeds of *O*. *minor*. These findings suggest that planteose is the main storage carbohydrate providing energy required for the early stages of germination of *O. minor* seeds. Plants generally store starch or lipids in seeds as a source of energy for germination. However, seeds of *S*. *hermonthica* lack detectable amount of starch (Vallance, 1951), suggesting that the dry seeds of root parasitic weeds may contain little or no starch. In *Striga asiatica* seeds, triacylglycerol were not hydrolysed during or after germination (Menetrez *et al.*, 1988), indicating that this species does not rely on lipids to provide energy for germination. The levels of starch and total fatty acids in GR24 treated *O. minor* seeds tended to increase through germination and these never fell below the levels in non-treated seeds (Fig. 3). These results indicate that starch and lipids are not significant energy sources for the seed germination of root parasitic weeds and support the hypothesis that planteose is the main storage compound for germination.

Planteose was also detected in the seeds of other root parasitic weeds in Orobanchaceae: *O. crenata*, *P. aegyptiaca*, and *S. hermonthica*. In all of these species, the amount of planteose in seeds decreased during germination (Fig. 4). Also, the change in sugar composition in seeds during germination was quite similar among the three broomrapes (*O. minor*, *O. crenata*, and *P. aegyptiaca*) (Fig. 4). These results suggest that sugar metabolism in germinating seeds is conserved among broomrapes. Together, these findings indicate that planteose may function as a storage carbohydrate for use during the early stages of seed germination of root parasitic weeds in Orobanchaceae.

In NJ-treated *O. minor* seeds at 7 DAG, a high accumulated level of sucrose was observed (Fig. 6), which suggests that NJ might inhibit sucrose degradation. The sum total of sucrose and planteose in the dry seeds (4.2 nmol·mg^-1^ fresh weight seed) was almost equal to the amount of sucrose (4.1 nmol·mg^-1^ fresh weight seed) in the NJ-treated seeds at 7 DAG. This result implies that the accumulated sucrose was probably a result of the inhibited degradation of the sucrose in the planteose metabolism and of that originally present in the dry seeds. Glucose recovered the germination rate in the presence of NJ (Fig. 7), indicating that glucose is required for seed germination of *O. minor*. There was significantly less glucose in NJ-treated *O. minor* seeds than in non-treated seeds. This decreased glucose level may have fatal effects on seed germination. There was also less fructose in NJ-treated seeds than in non-treated seeds. However, exogenous fructose could not fully recover the germination rate, suggesting that there is a much stronger requirement for glucose than for fructose during *O. minor* seed germination. The weak recovery of the germination rate by sucrose indicates that *O. minor* seeds are unable to metabolize sucrose in the presence of NJ.

In general, two classes of enzymes, INVs and SUSs, are involved in sucrose degradation. Both types of enzymes have important roles in plant development at diverse stages (Koch, 2004). Recent studies on *P*. *ramosa* showed that the transcript levels of *INV* and *SUS* genes and the activities of INVs increased in germinating seeds (Draie *et al.*, 2011; Péron *et al.*, 2012). Therefore, these two types of enzymes might have important roles in the germination of broomrape seeds. It is assumed that NJ affects the activities of INVs rather than SUSs because significant accumulation of sucrose was observed in NJ-treated seeds and the inhibition of germination was fully recovered only by adding exogenous glucose, which is a product of sucrose hydrolysis by INVs. In *O. minor* seeds, the activities of INVs gradually increased during germination, consistent with the results of previous studies on *P. ramosa*. NJ at 0.1mM did not inhibit the activities of INVs *in vitro*; however, at 5 DAG, the activities of INVs were significantly lower in NJ-treated (10 µM) seeds than in non-treated seeds (Fig. 8). Meanwhile, the activity of α-galactosidase was not changed in the presence of NJ. These results suggest that NJ specifically decreases the activities of INVs.

There should be other pathways producing monosaccharides during the late stages of germination because in the germinating seeds at 7 DAG, the amounts of monosaccharides were higher than the estimated amounts produced from sucrose and planteose degradation. Additionally, the level of monosaccharides was significantly increased from 5 to 7 DAG, whereas sucrose and planteose were almost consumed by 5 DAG (Fig. 2D–G). This increment might indicate the involvement of other pathways to produce monosaccharides (e.g. cell wall polysaccharide and/or fructan). It is known that sugars act as important signalling molecules like phytohormones throughout all stages of plant development (Rolland *et al.*, 2006; Eveland and Jackson, 2012). For example, as a sugar-inducible carbohydrate-related metabolism, a supply of sucrose, glucose, or fructose to *Arabidopsis* induces expression of a gene for β-amylase (Mita *et al.*, 1995). Because the substantial increase in monosaccharides was not observed in NJ-treated seeds at 7 DAG, it is likely that glucose provided from sucrose and planteose triggers the following processes required for germination, including the degradation of other storage products.

NJ is an iminosugar, and some other iminosugars have previously been reported to inhibit seedling growth. Seedling growth and root elongation in *Raphanus sativus* (kaiware radish) were inhibited by CS at 0.5mM or higher, and DNJ at 5mM or higher (Mega, 2004, 2005). Similarly, DNJ, *N*-butyl DNJ, and miglitol at 0.5mM inhibited root elongation in germinating barley (Stanley *et al.*, 2011). In both of those studies, the iminosugars did not inhibit seed germination. Also, the concentrations of iminosugars used in those studies were higher than those used in the present study (1–100 µM NJ for the inhibition of *O. minor* germination). At present, the MOA of these iminosugars remains unclear. In the case of *O. minor*, DNJ at 0.1mM inhibited radicle elongation (Supplementary Figure S6), but did not affect seed germination, as is the case in other plants (Supplementary Figure S5B). Because NJ inhibited *O. minor* seed germination at a much lower concentration, the inhibitory mechanism of NJ likely differs from those of other iminosugars such as DNJ. CS also inhibited the germination of *O. minor*, but higher concentrations of CS than NJ were required for the inhibitory effect. This finding suggests that NJ and CS inhibit germination via the same mechanism, but that they have different affinities for their target site(s). NJ did not inhibit seed germination in species other than *O. minor,* and only inhibited the root and radicle elongation of other species at high concentrations. Therefore, NJ may inhibit root elongation of these plants via a similar mechanism to that of other iminosugars.

Aoki and Hatanaka (1991) reported that 1,4-dideoxy-1,4-iminoarabinitol (DIA) at concentrations of 0.1–10mM inhibited growth of seedlings of rape (*Brassica napus*), lucerne (*Medicago sativa*), castor bean (*Ricinus communis*), barley (*Hordeum vulgare*), and rice (*Oryza sativa*), but had little effect on their seed germination. As in the present study, the activities of SAI and CWI were lower in 0.5mM DIA-treated lucerne seedlings than in non-treated seedlings. They also reported that DIA did not affect activities of acid INVs *in vitro*. Their findings, together with the results of this study, imply that NJ and DIA affect the activities of INVs indirectly; for example, by inhibiting transcriptional, translational, or post-translational processes. Recently, heterologous expression of tomato SAI (TIV-1) in *Pichia pastoris* revealed that *N*-glycosylation in TIV-1 was important for its activity and stability (Tauzin *et al.*, 2014). Considering the role of NJ as a glycosidase inhibitor, NJ may affect glycosylation of INVs in *O. minor*. However, at present, there is limited genomic or transcriptomic information available for *O*. *minor*. Therefore, further research is required to identify the target site of NJ.

NJ specifically inhibited germination of *O*. *minor* seeds, but not those of the other tested plants. This is believed to be the first report of broomrape-specific chemical inhibition of seed germination. The plant species could be ranked in terms of their sensitivity to NJ, from most sensitive to least sensitive, as follows: *O. minor* (obligate holoparasite), *S. hermonthica* (obligate hemiparasite), *P. japonicum* (facultative hemiparasite), spearmint (Lamiaceae), sesame (Pedaliaceae), and tomato (Solanaceae) (Supplementary Figure S8). Interestingly, this order is the reverse order of the evolutionary process of parasitism (Stevens, 2001; Westwood *et al.*, 2010). These results imply that the difference in the sensitivity to NJ among the tested plants is related to the divergence of sugar use during the evolutionary process of parasitism. In other words, sugar metabolism during germination may differ between broomrapes, parasitic plants in Orobanchaceae, and non-parasitic plants. In fact, sugar metabolism in germinating seeds of *S. hermonthica* may slightly differ from that in germinating seeds of broomrapes because there was less planteose in *S. hermonthica* seeds than in broomrape seeds (Fig. 4). In tomato, the sucrose content in seeds did not change during germination (Supplementary Figure S7C), while the sucrose content in seeds of root parasitic weeds decreased. The differences in sugar use during germination of root parasitic weeds in Orobanchaceae may be attributed to their ability to photosynthesize: hemiparasites are capable of fixing carbon themselves, whereas holoparasites obtain all of their reduced carbon from their hosts (Press *et al.*,1991; Irving and Cameron 2009). Because NJ inhibits only the germination of *O*. *minor*, some physiological events during its germination that are targeted by NJ may have arisen during evolution. Comparing the effects of NJ among a wide variety of plant species will clarify the relationship between NJ inhibition and the evolution of parasitism.

Finally, the results presented here show that planteose metabolism, including sucrose degradation, is essential during the early stage of germination of *O. minor* seeds. Chemical control of this metabolic pathway could provide new, specific methods to control root parasitic weeds, especially broomrapes. The results of this study further our understanding of the biochemistry of seed germination of root parasitic weeds. NJ can be used as a powerful inhibitor to clarify details of sugar metabolism during seed germination.

## Supplementary data

Supplementary data can be found at *JXB* online.

Table S1. ^1^H and ^13^C NMR spectral data of the trisaccharide.

Fig. S1. Purification of unknown trisaccharide from dry seeds of *O*. *minor*.

Fig. S2. GC-MS analysis of acid hydrolysates of sucrose, raffinose, and unknown trisaccharide.

Fig. S3. Structural identification of unknown trisaccharide by NMR.

Fig. S4. Effect of NJ on seed germination (A) and root elongation (B) of *P*. *japonicum*.

Fig. S5. Effects of glycosidase inhibitors on germination rate of *O. minor*.

Fig. S6. Effect of DNJ on radicle elongation of *O. minor* seeds.

Fig. S7. Effect of DNJ on germination, root elongation, and planteose

metabolism in planteose-containing seeds.

Fig. S8. Relationship between plant evolution and NJ effect.

## Funding

This study was partially supported by an Industrial Technology Research Grant Program from the New Energy and Industrial Technology Development Organization (KH, SM, and AO), a Grant-in-Aid for Young Scientists (A) from the Japan Society for the Promotion of Science (JSPS) (AO), a Research Fellowship for Young Scientist from JSPS (TW), and a Science and Technology Research Partnership for Sustainable Development from the Japan Science and Technology Agency and the Japan International Cooperation Agency (YS and AO).

## Supplementary Material

Supplementary Data

## Abbreviations:

CWI: cell wall invertase
CS: castanospermine
DAG: days after GR24 treatment
DGJ: 1-deoxygalactonojirimycin
DIA: 1,4-dideoxy-1,4-iminoarabinitol
DNJ: 1-deoxynojirimycin
ELSD, evaporative light scattering detector; GC-TOF-MS: Gas chromatography combined with time-of-flight mass spectrometry
HPLC, high-performance liquid chromatography; INV: invertase
MOA: mode of action
NJ: nojirimycin bisulfite
PC: principal component
PCA: principal component analysis
PVPP: polyvinylpolypyrrolidone
RFO: raffinose family oligosaccharides
SAI: soluble acid invertase
SNI: soluble neutral invertase
SUS: sucrose synthase
UDP: uridine diphosphate; UPLC, ultra-performance liquid chromatography.
