# Supplementary material for: Planteose as a storage carbohydrate required for early stage of germination of Orobanche minor and its metabolism as a possible target for selective control
Source: J Exp Bot. 2015 Mar 28;66(11):3085–97. doi: 10.1093/jxb/erv116 (PMC4449533; doi:10.1093/jxb/erv116)
Supplement: Supplementary Data [file supp_66_11_3085__index.html]

Planteose as a storage carbohydrate required for early stage of germination of Orobanche minor and its metabolism as a possible target for selective control — Planteose as a storage carbohydrate required for early stage of germination of Orobanche minor and its metabolism as a possible target for selective control — Supplementary Data 

# Planteose as a storage carbohydrate required for early stage of germination of *Orobanche minor* and its metabolism as a possible target for selective control

## Supplementary Data

Data files

**Files in this Data Supplement:**

- Supplementary Data - Supplementary Data
