# Supplementary material for: Planteose as a storage carbohydrate required for early stage of germination of Orobanche minor and its metabolism as a possible target for selective control
Source: J Exp Bot. 2015 Mar 28;66(11):3085–97. doi: 10.1093/jxb/erv116 (PMC4449533; doi:10.1093/jxb/erv116)
Supplement: Supplementary Data [file supp_erv116_jexbot137869_file001.pdf]

Journal of Experimental Botany

Title:

Planteose as a storage carbohydrate required for early germination process of *Orobanche minor* and its metabolism as a possible target for selective control

Authors:

Takatoshi Wakabayashi, Benesh Joseph, Shuhei Yasumoto, Tomoyoshi Akashi, Toshio Aoki, Kazuo Harada, Satoru Muranaka, Takeshi Bamba, Eiichiro Fukusaki, Yasutomo Takeuchi, Koichi Yoneyama, Toshiya Muranaka, Yukihiro Sugimoto and Atsushi Okazawa

Supplementary Table S1 ^{1}H and ^{13}C NMR spectral data of the trisaccharide.

		δ_C	δ_H		J_{HH}
Glc	1	94.7	5.44	*d*	4.0
	2	74.0	3.56	*dd*	10.0, 3.9
	3	75.5	3.76	*dd*	10.2, 8.7
	4	72.4	3.44	*dd*	9.8, 9.0
	5	75.2	3.86	*ddd*	9.8, 3.7, 1.5
	6	63.3	3.86	*dd*	13.0, 2.3
			3.78	*m*	
Fru	1	64.4	3.68	*d*	4.1
	2	106.7			
	3	78.9	4.22	*d*	8.5
	4	77.2	4.10	*t*	8.5
	5	82.3	4.06	*m*	
	6	71.7	3.78	*m*	
			4.06	*m*	
Gal	1	101.4	5.00	*d*	3.9
	2	71.2	3.84	*dd*	10.4, 3.8
	3	72.2	3.91	*dd*	10.4, 3.2
	4	72.0	4.03	*dd*	3.4, 1.3
	5	73.9	3.99	*m*	
	6	63.9	3.75	*m*	

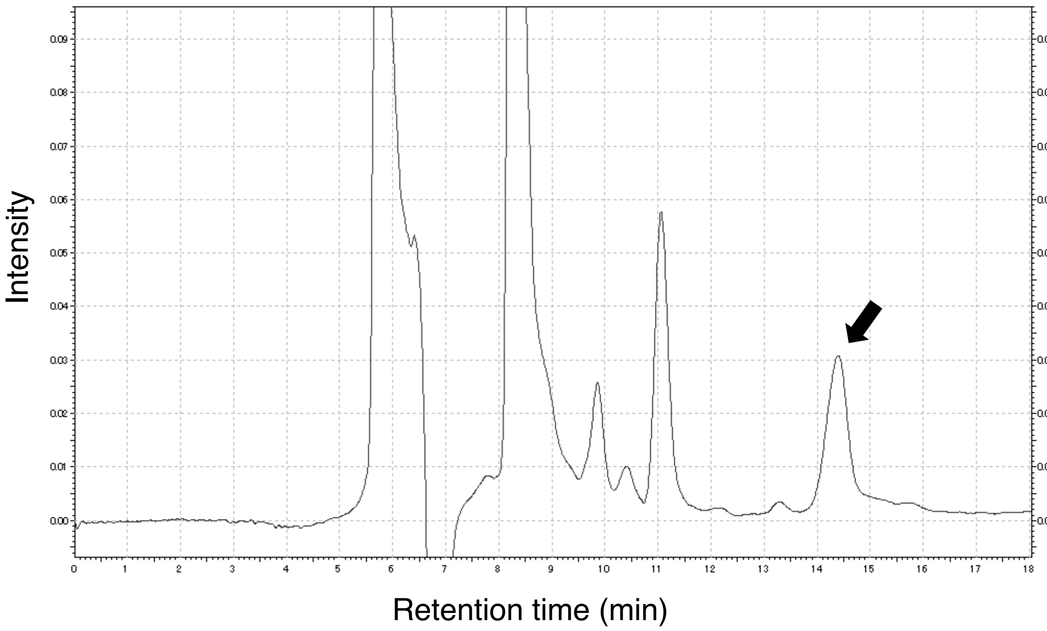

Supplementary Figure S1. Purification of unknown trisaccharide from dry seeds of *O. minor*. Sugars were extracted from dry seeds of *O.* minor; unknown trisaccharide was purified from seed extract by HPLC. Arrow marks unknown trisaccharide.

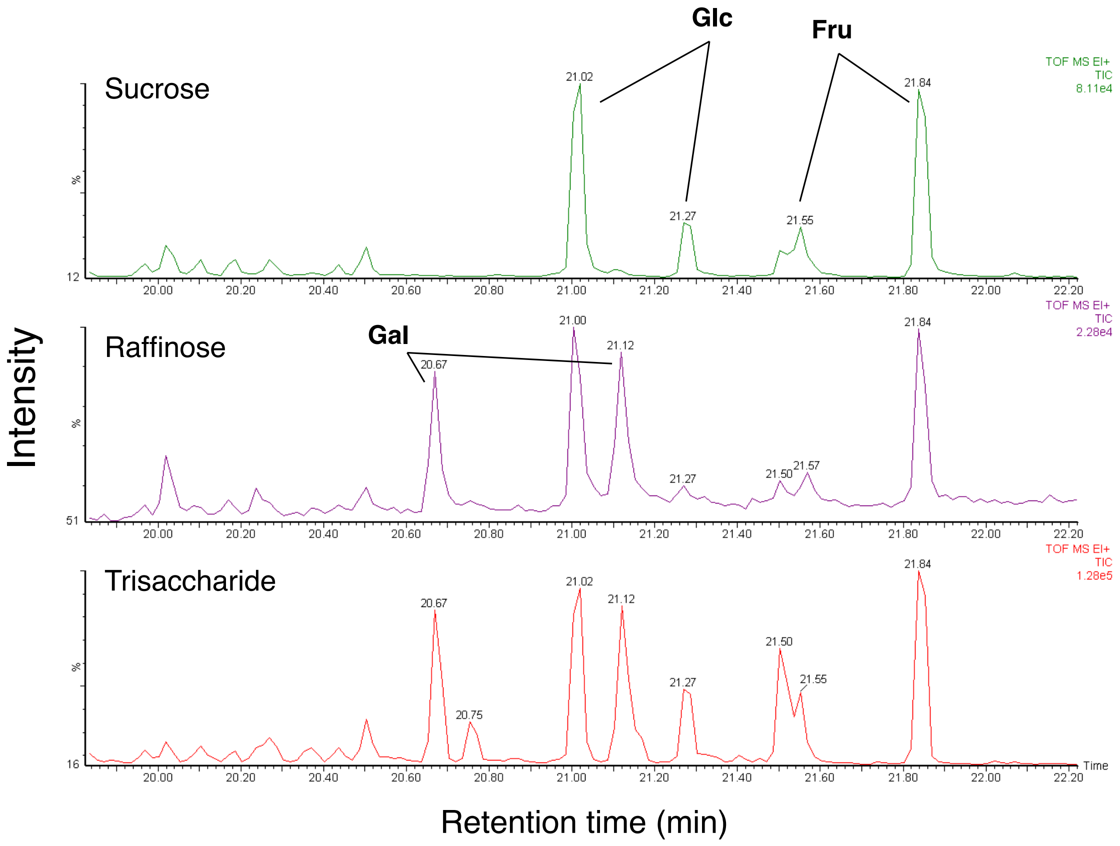

Supplementary Figure S2. GC-MS analysis of acid hydrolysates of sucrose, raffinose, and unknown trisaccharide. Sucrose, raffinose, and purified trisaccharide were acid hydrolyzed in 1 N HCl at 95°C for 1 h. Monosaccharides released from trisaccharide by acid hydrolysis were consistent with those released from raffinose. Glc, glucose; Fru, fructose; Gal, galactose.

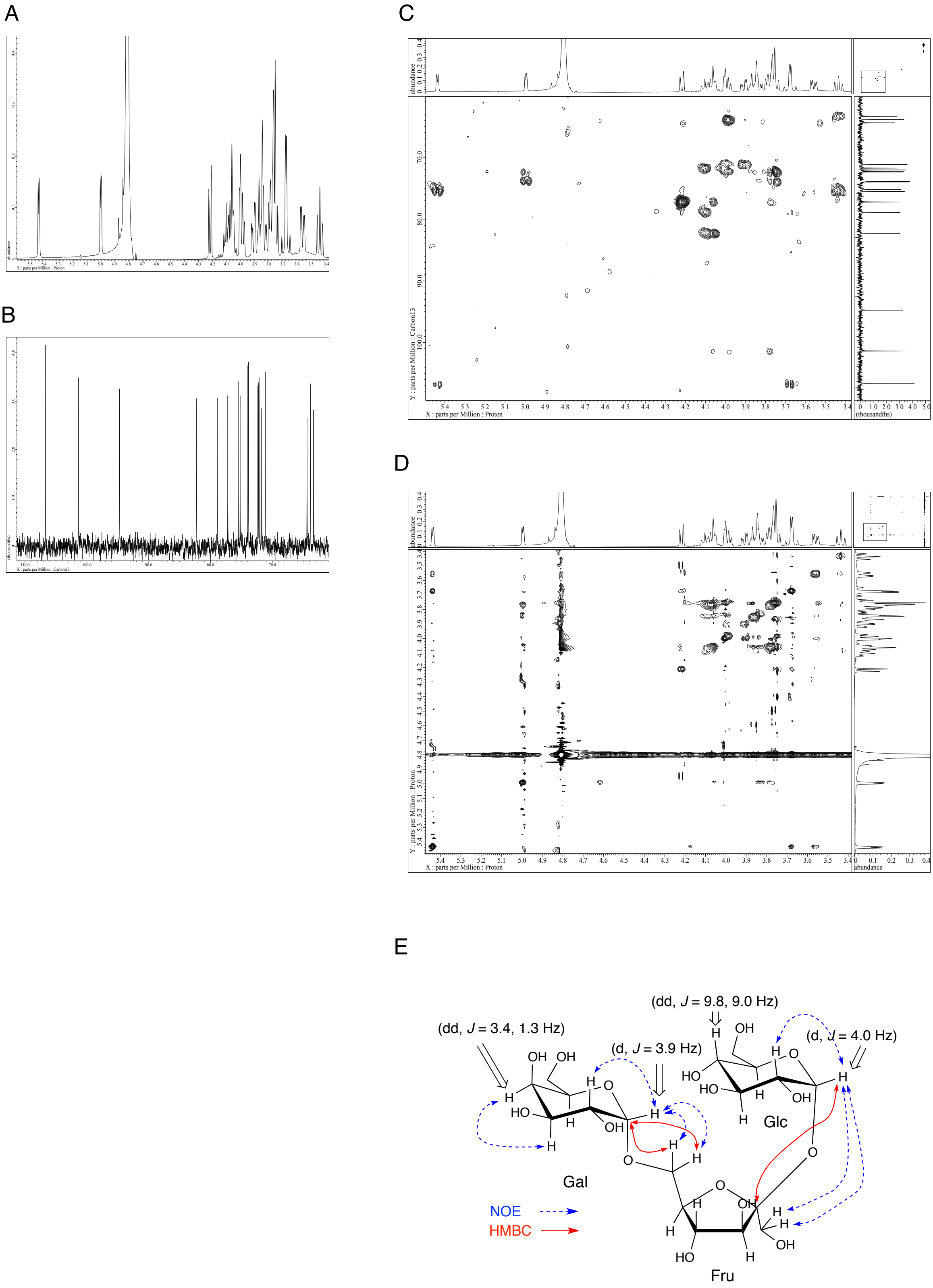
A
B
C
D
E
X : parts per Million : Proton
X : parts per Million : Carbon13
Y : parts per Million : Carbon13
Y : parts per Million : Proton
abundance
(thousandths)
(dd, J = 3.4, 1.3 Hz)
(d, J = 3.9 Hz)
(dd, J = 9.8, 9.0 Hz)
(d, J = 4.0 Hz)
Gal
Glc
Fru
NOE
HMBC

Supplementary Figure S3. Structural identification of unknown trisaccharide by NMR. (A) ^{1}H and (B) ^{13}C NMR signals. (C) HMBC and (D) NOESY spectra of trisaccharide. (E) Structure of identified trisaccharide, planteose (α-D-galactopyranosyl-(1→6)-β-D-fructofuranosyl-(2→1)-α-D-glucopyranoside) in which C1 of galactose is attached to C6 of fructose moiety of sucrose via an α-glycosidic linkage. Figure shows selected long range correlations observed in HMBC spectrum and NOE of planteose.

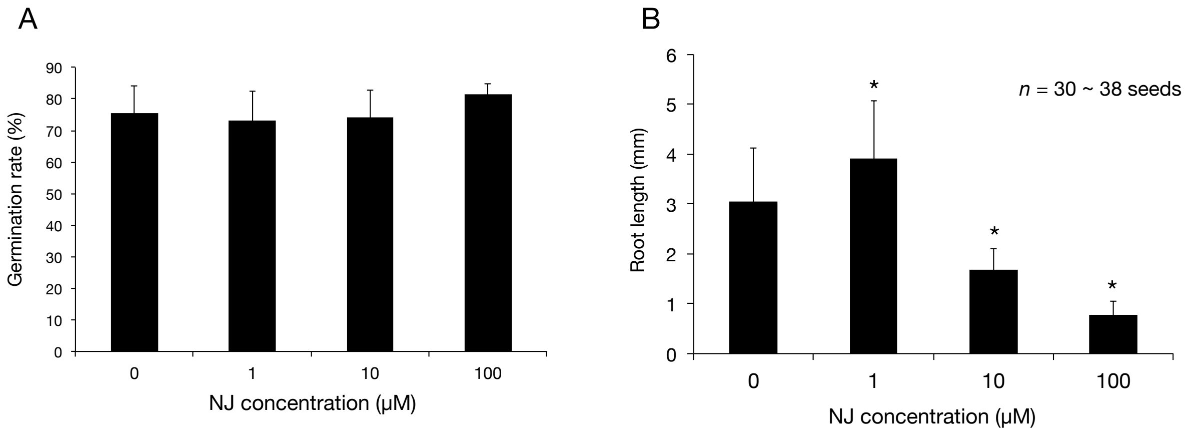

Figure S4. Effect of NJ on seed germination (a) and root elongation (b) of *P. japonicum*. (A) NJ, even at 100 μM, did not affect germination rate of *P. japonicum*. Number of germinated seeds (out of 20) was counted (mean ± SD, $n = 3$). (B) Effects of NJ on root elongation became visible at 10 μM; root length of seedlings treated with 100 μM NJ was approximately one-quarter that of control root length. Radicle lengths of 30 to 38 germinated seeds were measured using ImageJ software. Asterisks indicate significant differences in the root lengths between control and NJ-treated seeds ($P < 0.05$, Student's *t* test).

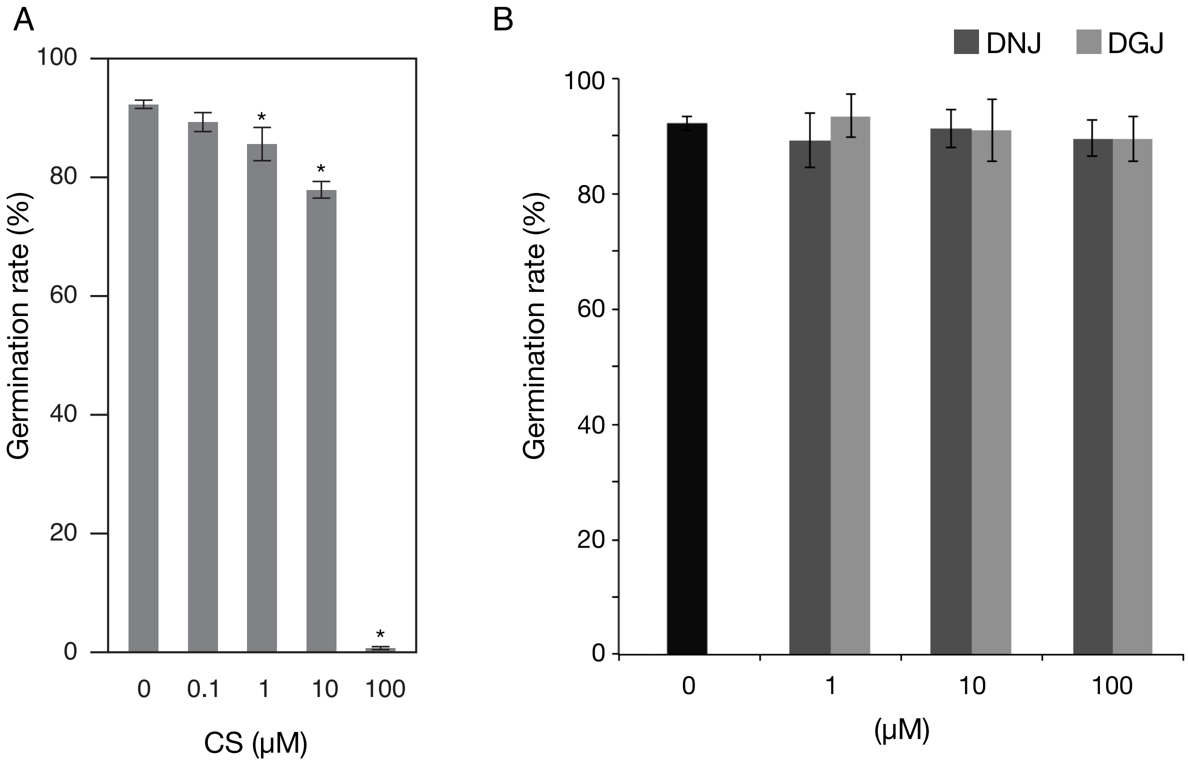

Supplementary Figure S5. Effects of glycosidase inhibitors on germination rate of *O. minor*. Germination rates of *O. minor* seeds in the presence of various concentrations of CS (A) and DNJ and DGJ (B). Number of germinated seeds (out of 200) was counted (mean ± SD, n = 3). Asterisks indicate significant differences in the root lengths between control and glycosidase inhibitor-treated seeds ($P < 0.05$, Student's t test).

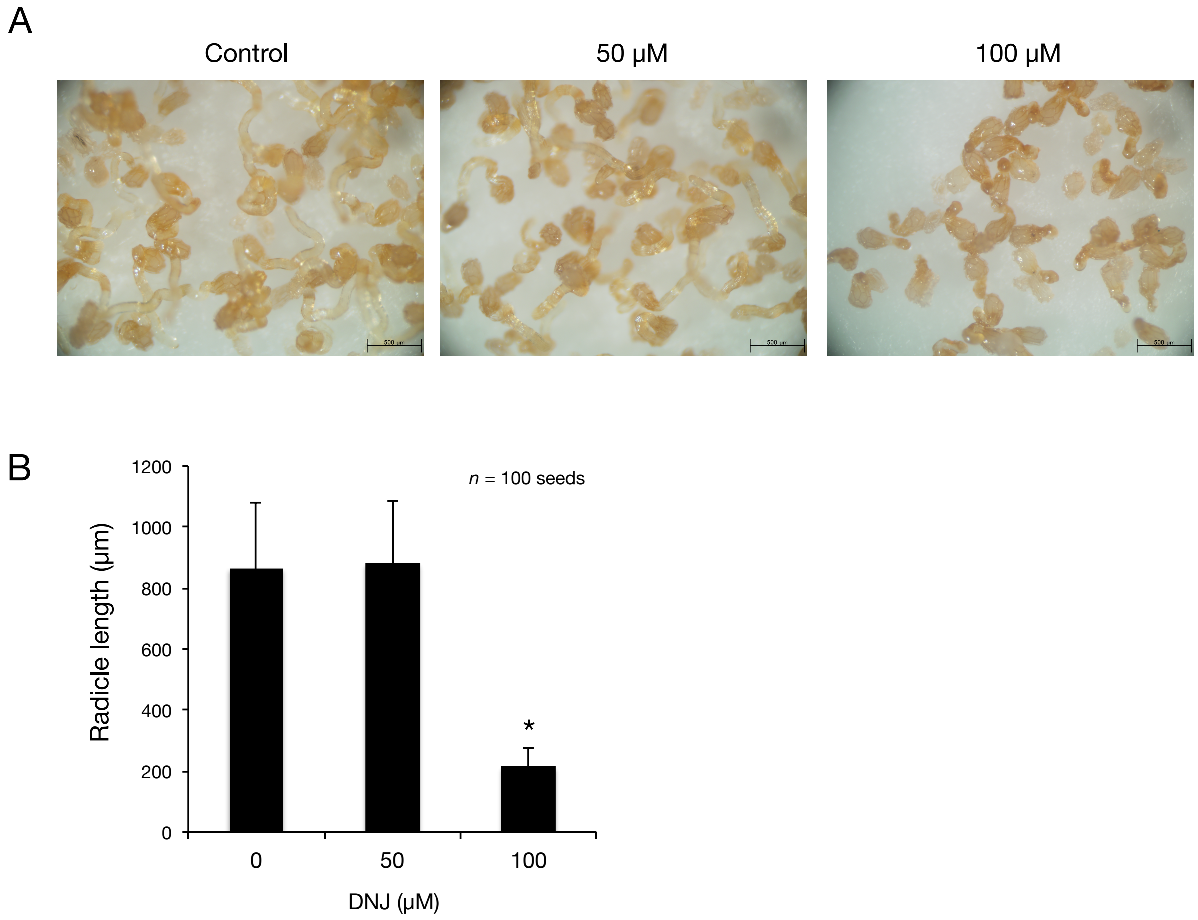

Supplementary Figure S6. Effect of DNJ on radicle elongation of *O. minor* seeds. Conditioning period was 7 days; 10 mg·l^{-1} (w/v) GR24 with or without DNJ was applied on final day of conditioning. Effect of DNJ was observed at 7 days after GR24 treatment. (A) Images of control seeds (left), 50 μM DNJ-treated seeds (middle), and 100 μM DNJ-treated seeds (right). Bar: 500 μm. (B) Radicle lengths of *O. minor* treated with DNJ. Radicle lengths of 100 germinating seeds were determined using ImageJ software. The radicle length treated with 100 μM DNJ was approximately one-quarter that of control radicle length. Asterisk indicates significant difference in the root lengths between control and DNJ-treated seeds ($P < 0.05$, Student's t test).

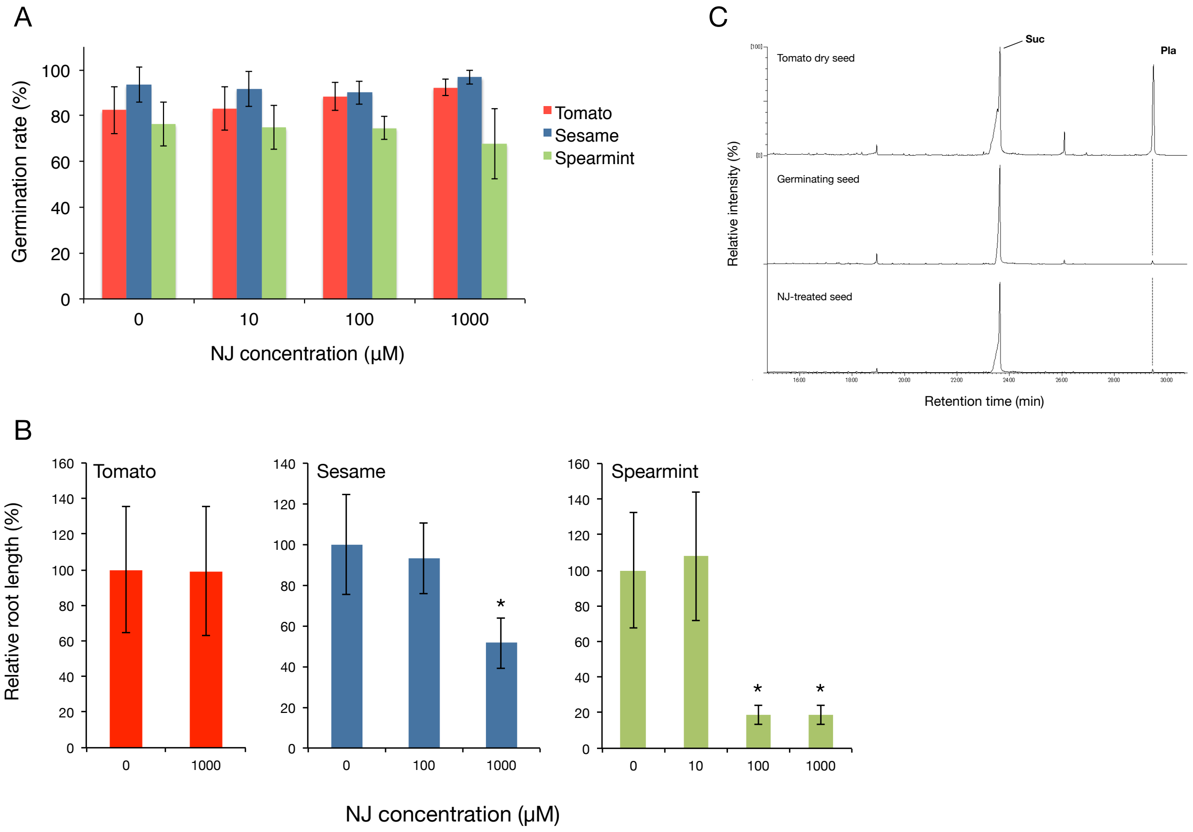

Supplementary Figure S7. Effect of NJ on germination, root elongation, and planteose metabolism in planteose-containing seeds. (A) Effect of NJ on germination of seeds containing planteose. Number of germinated seeds (out of 20) was counted. Analysis was carried out in triplicate (mean ± SD, $n = 3$). (B) Effects of NJ on root elongation. Germinated seeds were selected and radicle lengths were determined using ImageJ software. Numbers of seeds counted were as follows: 22 (control) and 29 (1 mM NJ) for tomato; 29 (control), 31 (100 µM NJ), and 21 (1 mM NJ) for sesame; and 24 (control), 26 (10 µM NJ), 33 (100 µM NJ), and 33 (1 mM NJ) for spearmint. Asterisks indicate significant differences in the root lengths between control and NJ-treated seeds ($P <$ 0.05, Student's t test). (C) GC-MS analyses of sugar composition in dry, germinating, and NJ-treated tomato seeds. There were no differences in sugar composition between

non-treated germinated seeds and NJ-treated seed

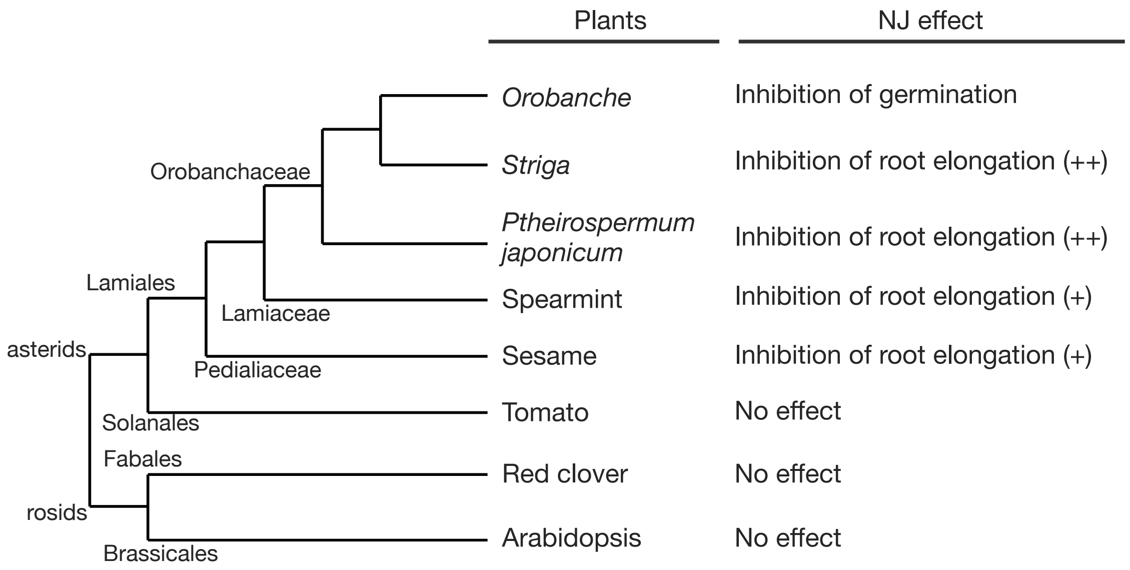

Supplementary Figure S8. Relationship between plant evolution and NJ effect. The plant species is closer to *O. minor*, the stronger the effect of NJ appeared. (++) indicates strong inhibitory effect; (+) indicates weak inhibitory effect.
